# A gene expression comparison of *Trypanosoma brucei* and *Trypanosoma congolense* in the bloodstream of the mammalian host reveals species-specific adaptations to density-dependent development

**DOI:** 10.1371/journal.pntd.0006863

**Published:** 2018-10-11

**Authors:** Eleanor Silvester, Alasdair Ivens, Keith R. Matthews

**Affiliations:** Institute for Immunology and Infection Research, School of Biological Sciences, University of Edinburgh, Edinburgh, United Kingdom; Universiteit Antwerpen, BELGIUM

## Abstract

In the bloodstream of mammalian hosts *Trypanosoma brucei* undergoes well-characterised density-dependent growth control and developmental adaptation for transmission. This involves the differentiation from proliferative, morphologically ‘slender’ forms to quiescent ‘stumpy’ forms that preferentially infect the tsetse fly vector. Another important livestock trypanosome, *Trypanosoma congolense*, also undergoes density-dependent cell-cycle arrest although this is not linked to obvious morphological transformation. Here we have compared the gene expression profile of *T*. *brucei* and *T*. *congolense* during the ascending phase of the parasitaemia and at peak parasitaemia in mice, analysing species and developmental differences between proliferating and cell-cycle arrested forms. Despite underlying conservation of their quorum sensing signalling pathway, each species exhibits distinct profiles of gene regulation when analysed by orthogroup and cell surface phylome profiling. This analysis of peak parasitaemia *T*. *congolense* provides the first molecular signatures of potential developmental competence, assisting life cycle developmental studies in these important livestock parasites. Furthermore, comparison with *T*. *brucei* identifies candidate molecules from each species that may be important for their survival in the mammalian host, transmission or distinct tropism in the tsetse vector.

## Author summary

Animal African trypanosomiases are important diseases of livestock in sub-Saharan Africa. Two of the responsible parasite species are *Trypanosoma brucei* and *Trypanosoma congolense*, both being blood-borne parasites transmitted by tsetse flies. In *T*. *brucei* there is a well-characterised developmental event in the bloodstream that prepares the parasite for tsetse transmission—the generation of morphologically stumpy forms. In contrast, *Trypanosoma congolense* does not undergo the same obvious morphological event, but does respond to parasite density in the mammalian bloodstream by accumulating as a cell cycle arrested form. This prompted us to explore the adaptations of *T*. *congolense* in response to cell density in blood and to compare this with *T*. *brucei*. The datasets generated, and their analysis, represent a first detailed transcriptional profile for *T*. *congolense* and also a new high-resolution analysis of the developmental forms of *T*. *brucei* in a mammalian host. Critically, the analysis also carefully characterised the biological material used for RNA-seq analysis with respect to cell cycle status, morphology and the expression (in the case of *T*. *brucei*) of PAD1 –a molecular marker for stumpy forms. The manuscript highlights clear differences in the developmental adaptation of each parasite species, with *T*. *congolense* showing less extreme adaptation at peak parasitaemia than *T*. *brucei*. Nonetheless, several predicted surface protein families in *T*. *congolense* are strongly upregulated at high parasite density in the bloodstream, which may represent adaptations for their transmission or survival.

## Introduction

African trypanosomes are responsible for Human African Trypanosomiasis (HAT) and Animal African Trypanosomiasis (AAT) in sub Saharan Africa[1]. The human disease is caused by two sub-species of *Trypanosoma brucei*, *T*. *b*. *rhodesiense* and *T*. *b*. *gambiense*, whilst *Trypanosoma brucei brucei* is responsible only for animal disease because it is sensitive to the trypanolytic component of human serum[2]. However, *T*. *b*. *brucei* is not the major cause of AAT, this being predominantly caused by infection with two alternative trypanosome species, *Trypanosoma congolense* and *Trypanosoma vivax*[3]. In combination, AAT threatens approximately 55 million cattle in sub-Saharan Africa and despite considerable costs to agriculture of prophylaxis against trypanosomiasis, approximately 3 million animals succumb each year[4]. *Trypanosoma congolense* is considered the most important agent of disease, with *Trypanosoma brucei brucei* comprising only a minority of infections.

Although African trypanosomiasis is caused by different trypanosome species, these species are all transmitted by the tsetse fly[5]. This arthropod disease vector transmits the parasites by feeding on the blood of infected mammalian hosts, after which the trypanosomes undergo life cycle development in the fly before being transmitted to a new mammalian host. Although all three trypanosome species (*T*. *brucei*, *T*. *congolense*, *T*. *vivax*) have tsetse flies as their vector, the developmental progression of the different parasites differs within the arthropod[6]. For *Trypanosoma brucei*, the parasites enter the midgut, where they multiply as procyclic forms, before migration to the salivary glands where attached epimastigote and then metacyclic forms develop, the latter being mammal infective. For *Trypanosoma congolense*, there is also multiplication in the fly midgut as procyclic forms before the parasites develop in to long trypomastigotes and migrate via the proventriculus and foregut to the proboscis and cibarium where the transition to epimastigote forms and then metacyclic forms occurs [7]. Contrasting with the other trypanosome species, *Trypanosoma vivax* does not develop in the fly midgut, but rather matures in the proboscis forming epimastigote and infective metacyclic forms in the mouthparts. As a consequence of this less elaborate developmental path, *Trypanosoma vivax* can also exhibit mechanical transmission by tsetse flies and other biting arthropods, this having assisted the spread of this parasite outside the tsetse belt and in South America [3].

For *Trypanosoma brucei* and *Trypanosoma congolense*, the development of the parasite within the tsetse fly vector is characterised by their expression of stage-specific surface proteins, with EP and GPEET procyclin characterising early and late procyclic forms in the tsetse midgut for *T*. *brucei*[8], and epimastigote forms expressing the BARP surface coat[9]. For *T*. *congolense*, GARP was originally described as a marker for the midgut forms[10], but subsequent analysis has identified a procyclin-related protein encoded in the *T*. *congolense* genome which seems to be functionally equivalent to EP/GPEET procyclin[11, 12] and grouped with *T*. *brucei* procyclins in a cell surface phylome analysis (i.e. the evolutionary relatedness between predicted cell surface proteins in different trypanosome species).

One characteristic of *Trypanosoma brucei* is that it exhibits clear developmental preadaptation for transmission in the mammalian host. Specifically, the parasite proliferates as a morphologically slender form until parasite numbers increase, at which point a density dependent quorum sensing (QS) mechanism causes differentiation to a non-proliferative, stumpy form[13]. The stumpy form is characterised by altered morphology[14], cell cycle arrest in G1/G0[15, 16], a reduced anterior free flagellum, expression of the stumpy specific protein PAD1 [17]and appearance of an elaborated mitochondrion. The latter represents the preparation for metabolic adaptation of the parasite to the tsetse midgut, since in the bloodstream energy generation is via the use of blood glucose in glycolysis, whereas in the vector oxidative phosphorylation and the utilisation of proline as an energy source predominates[18, 19]. Critically, stumpy forms are adapted for transmission[20, 21], exhibiting efficient and synchronous differentiation to procyclic forms when exposed to a cis-aconitate, mild acid or protease stimulus, of which the latter two conditions are lethal to slender forms[22]. The generation of transmissible stumpy forms in the bloodstream is stimulated by a soluble stumpy induction factor (SIF)[23] that is currently unidentified, although the signalling pathway through which the signal is transduced in the trypanosome has been characterised in some detail[24, 25].

Whilst the production of stumpy forms in *T*. *brucei* is characteristic of that species, *Trypanosoma congolense* has also been shown to undergo a related phenomenon albeit without obvious morphological transformation[26]. Thus, *T*. *congolense* in the mammalian bloodstream exhibit proliferation control in response to parasite numbers, accumulating in G1/G0 when parasite numbers exceed approximately 8x10^7^/ml. Their genome also encodes orthologues of *T*. *brucei* QS signalling pathway components, and at least one of these (TcHYP2; *Tc*IL3000.0.19510) can complement a *T*. *brucei* null mutant to restore stumpy formation in that species. This raises the question of whether there is a distinct developmental form of *Trypanosoma congolense* equivalent to the stumpy form of *T*. *brucei*. Here, we have analysed the transcriptome of *T*. *congolense* in the ascending phase of its parasitaemia and at the peak of parasitaemia when the proliferation of the parasites is reduced by an accumulation in G1/G0. We have then compared these with the transcriptomes of *T*. *brucei* slender and stumpy forms, respectively. This has defined molecular characteristics of the peak parasitaemia *T*. *congolense* forms and highlighted molecules that may adapt the parasite for chronicity in the bloodstream or preadapt the parasite for its developmental path in the tsetse fly. The identification of enriched molecules at the peak of parasitaemia may provide morphology-independent markers for a possible transmissible form of *T*. *congolense*.

## Methods

### Ethics statement

All animal experiments were carried out after local ethical approval at the University of Edinburgh Animal Welfare and Ethical Review Body (approval number PL02-12) and were approved under the United Kingdom Government Home office licence P262AE604 to satisfy requirements of the United Kingdom Animals (Scientific Procedures) Act 1986.

### Trypanosomes

*T*. *congolense* IL3000 and *T*. *brucei* EATRO 1125 AnTat1.1. 90:13 parasites were used for infections. *T*. *congolense* IL3000 was derived from the ILC-49 strain that was isolated from a cow in the Trans Mara, Kenya [27]. *T*. *brucei* EATRO 1125 AnTat1.1. 90:13 were provided by Professor Markus Engstler and Professor Michael Boshart. For generation of *T*. *b*. *brucei* RNA-seq samples, mice were treated with 25mg/ml cyclophosphamide at least two hours prior to infection. For generation of *T*. *congolense* RNA-seq samples cyclophosphamide was not used.

Infections were usually monitored daily from day 3 post-infection by tail snip. On the final day of an experiment, total blood was harvested from mice by cardiac puncture using a Microlance 0.6 x 25mm needle and a 2ml syringe containing 250μl of 2% sodium citrate. Trypanosomes were purified from whole blood by passage through a DE52 column (Whatman anion exchange cellulose, Z742600) at pH 7.8.

### Cell cycle analyses

Cells were smeared onto slides, left to dry and fixed in cold methanol, and these slides were used for cell cycle analysis. Slides were initially rehydrated for 5 minutes in 1x PBS then 30μl of a DAPI working dilution (10μg/ml in PBS) was applied to the smears. Slides were incubated in a humidity chamber for 2 minutes and were then washed for 5 minutes in 1x PBS. Slides were then mounted with 40μl Mowiol containing 2.5% DABCO (1, 4-diazabicyclo[2.2.2]octane) and were left to dry overnight at room temperature in the dark, before storage at 4°C. Slides were analysed on a Zeiss Axioskop 2 plus for KN configuration counts, and QCapture software (QImaging) was used for image capture.

### RNA isolation and northern blotting

RNA samples were prepared using the Qiagen RNeasy kit (Qiagen, 74106) according to the manufacturer’s instructions. Briefly no more than 5x10^7^ cells were pelleted and resuspended in 594μl RLT buffer with 6μl β-mercaptoethanol. Samples were stored at -80°C before processing according to the protocol ‘Purification of Total RNA from Animal cells using spin technology’. RNA samples were resuspended in 10μl RNase free water per 10^7^ cells used to prepare the sample. The concentration and purity of the resultant RNA was measured on a Nanodrop spectrophotometer. RNA samples were stored at -80°C.

Northern blotting was carried out using digoxigenin labelled riboprobes as described in [28].

### Protein analyses

Protein samples were prepared and analysed for PAD1 expression according to[28].

### Bioinformatics

RNA-Seq was carried out by BGI Hong Kong using a Truseq library preparation protocol with polyA selection, and sequenced on a HiSeq2000 platform with 90bp paired end reads. Quality control on generated data was performed using Fast QC v0.10.0 (http://www.bioinformatics.babraham.ac.uk/projects/fastqc/) and paired end reads were trimmed using cutadapt v1.9.1 [ref: https://cutadapt.readthedocs.io/en/stable/] before alignment of the reads to the *T*. *congolense* genome (ftp://ftp.sanger.ac.uk/pub/project/pathogens/gff3/CURRENT/, with sequences and CDS gene model coordinates obtained 18 February 2015), or *T*. *brucei* genome (ftp://ftp.sanger.ac.uk/pub/project/pathogens/gff3/CURRENT/, with sequences and CDS gene model coordinates obtained 18 February 2015). Alignment was carried out using Bowtie 2 v2.2.7 (http://bowtie-bio.sourceforge.net/bowtie2/index.shtml [29], and was not restricted to annotated genes. Read counts were normalised to reads per kb/ map (rpkm), in order to account for gene size and the different read depths of the replicates. A joint quantile threshold cut-off of 10% was applied across the relevant replicate rpkms to remove the lowest numbers of reads from each group (e.g. ascending, peak). A group-wise comparison using the R/Bioconductor software package limma [30]was then performed between the ascending parasitaemia and peak parasitaemia replicates; significance values were corrected for multiple testing (adjusted P values) using eBayes. Phylome data were as described by[11]; the family member sets were sourced from http://www.genedb.org/Page/trypanosoma_surface_phylome.

OrthoMCL [31] clusters were obtained from GeneDB [in 2015]. The “Trypanosom**A**” ortho group was generated using: *T*. *cruzi*, *T*. *vivax*, *T*. *congolense*, *T*. *brucei 927 and T*. *brucei gambiense*.

The “Trypanosom**E**” ortho group was generated using: *T*. *cruzi*, *T*. *vivax*, *T*. *congolense*, *T*. *brucei* 927, *T*. *brucei gambiense*, *L*. *braziliensis*, *L*. *major*, *L*. *infantum*, *L*. *donovani*, *L*. *mexicana*.

### Data deposition

Data is available at GEO via the link: https://www.ncbi.nlm.nih.gov/geo/query/acc.cgi?acc=GSE114813.

### Production of figures

All graphs were produced using GraphPad Prism version 6 (GraphPad Software, La Jolla, California, USA, www.graphpad.com). Microscope images were generated (i.e. cropping, brightness, contrast, overlay, scale bar application) using Image J 64 [32].

## Results

**Fig 1:**
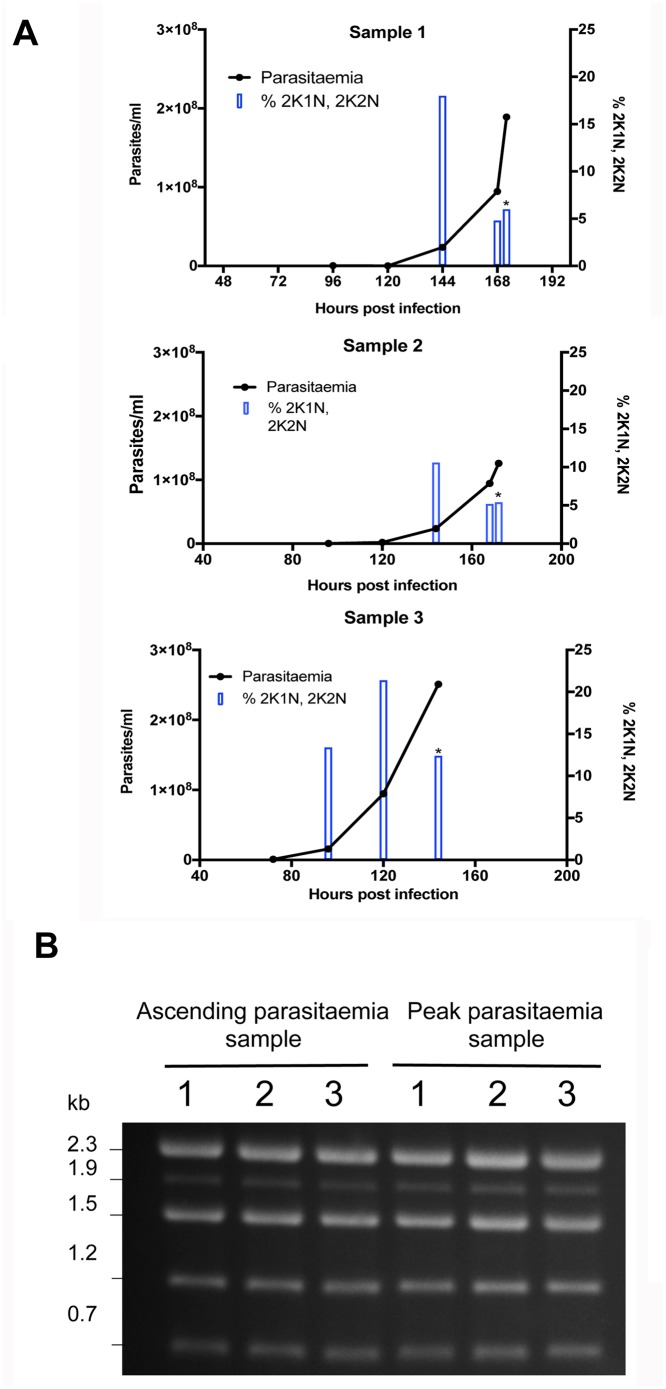
Biological characterisation of *T*. *congolense* material for expression analysis. (A) Parasitaemia (lines) and cell cycle status (bars) of *T*. *congolense* IL3000 in murine infections used to generate samples for ‘peak parasitaemia’ transcriptome analysis. The proportion of parasites presenting 2 kinetoplast, 1 nucleus (2K1N), or 2 kinetoplast 2 nuclei (2K2N) configurations was assessed in 500 cells at each time point, providing a measure of proliferating cells in the population. As the parasitaemias progressed beyond 1x10^8^ parasites/ml the proportion of proliferating parasites declined indicative of cell cycle arrest. Asterisks indicate when the sample was harvested. (B) Ethidium bromide stained total RNA of *T*. *congolense* ‘ascending’ and ‘peak’ parasitaemia samples. Note that *T*. *congolense* presents 5 major rRNA bands, contrasting with 3 in *T*. *brucei*.

We sought to analyse the RNA expression profile of *T*. *congolense* derived from infections in mice, isolated either during the ascending phase of infection within the first wave of parasitaemia, or at the peak of that wave. In *T*. *congolense*, high parasitaemia is linked to density-dependent cell cycle arrest, similar to the quorum sensing response of *T*. *brucei* during the transition from slender to stumpy forms. Therefore, to validate the biological characteristics of material isolated from infections, we determined the proportion of parasites with 1 kinetoplast and 1 nucleus (G1 and G0-arrested cells), 2 kinetoplasts and 1 nucleus (G2 phase cells and early mitotic cells) and 2 kinetoplasts and 2 nuclei (mitotic and post mitotic cells). Fig 1A shows the infection profile of 3 mice over 6 or 7 days. As observed previously [28], there was a reduction in the proportion of proliferating cells (2K1N, 2K2N) accompanying peak parasitaemia (>1x10^8^/ml) relative to when the parasitaemia was ascending, consistent with density-dependent cell cycle arrest. Parasites were then harvested from these infections on day 6 or 7 post-infection and RNA prepared after their purification, providing ‘peak parasitaemia’ samples. To recover sufficient material to analyse by RNA-Seq in the ascending phase of parasitaemia, it was necessary to combine parasite material from more than 1 mouse infection per replicate, such that 3 pooled but independent RNA samples generated from a total of 8 mouse infections were isolated. In each case, the material was derived from day 5 of infection, harvested from parasites exhibiting a mean of 12% 2K1N and 2K2N cells (range 9–16%), when mean parasite numbers were 5.4x10^7^/ml (with a range of 2.4x10^7^-9.5 x10^7^/ml) (the parasitaemias and associated cell cycle data for each sample are shown in S1 Fig). Fig 1B shows the profile of ribosomal RNAs from the three replicate ascending and peak parasitaemia populations, demonstrating the 5 rRNA bands characteristic of *T*. *congolense* and confirming integrity of the isolated RNA samples from each replicate and each phase of infection.

**Fig 2:**
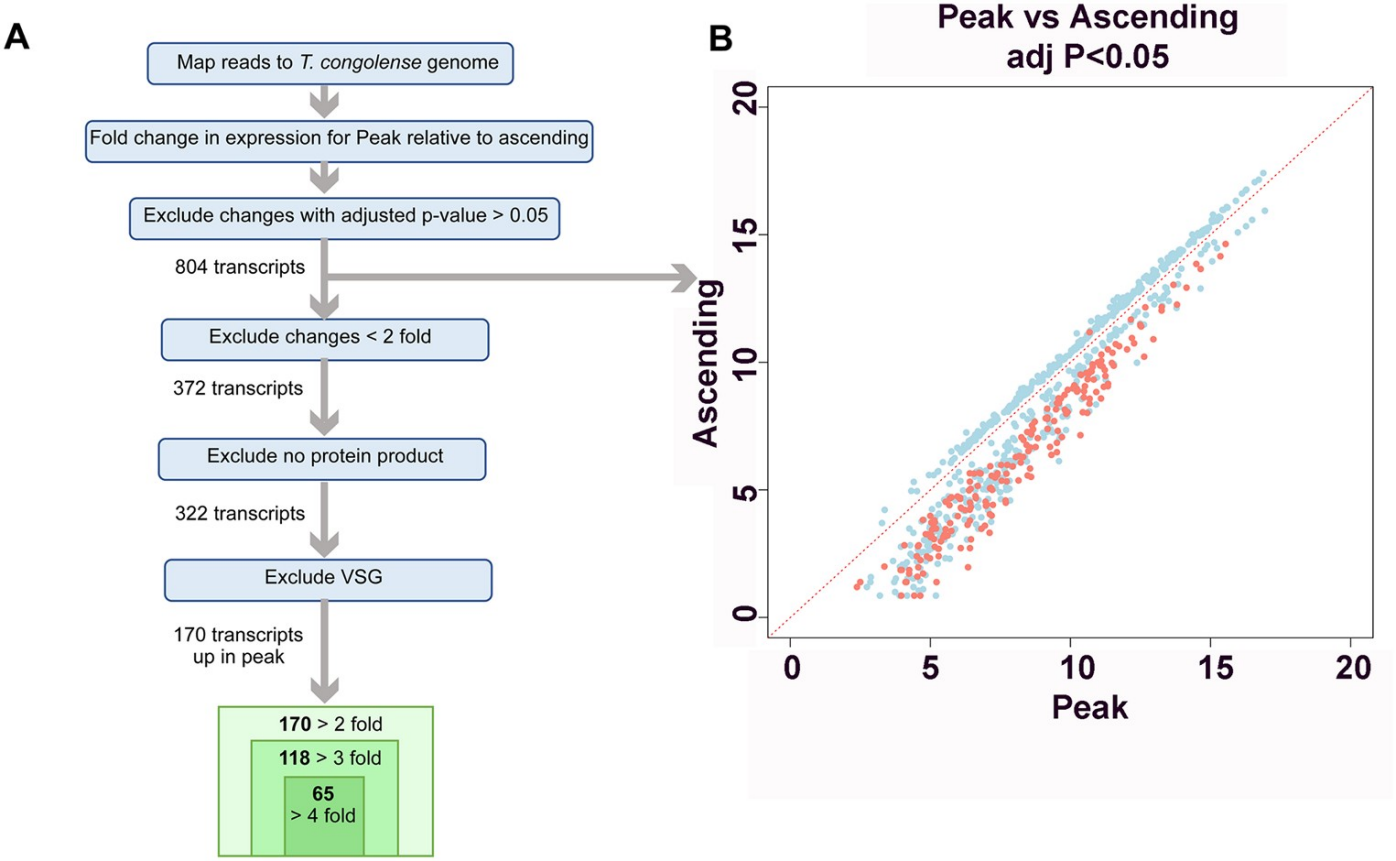
Expression differences between ascending and peak parasitaemia *T*. *congolense*. *(A*.*)* Strategy for the comparison of mRNA expression between ascending and peak parasitaemia *T*. *congolense*. (B.)Scatterplot depicting transcripts that show significant (adjusted p value <0.05) expression difference between ascending and peak parasitaemia. Transcripts annotated as VSG are coloured red, these being significantly upregulated in the peak parasitaemia samples.

The *T*. *congolense* RNA samples were then subjected to RNA-Seq profiling. S1 Table reports for each transcript the detected fold change (FC) between peak and ascending replicates as Log2FC, with positive values representing transcripts more abundant at peak parasitaemia than during ascending parasitaemia, whereas negative values indicate transcripts less abundant at peak parasitaemia than during ascending parasitaemia. When an adjusted p-value cut-off of p<0.05 was applied, 804 transcripts were found to have significant changes in abundance in peak relative to ascending data sets (Fig 2A). Average transcript levels at peak versus ascending parasitaemia for each of the 804 transcripts with significant changes were plotted (Fig 2B). Notably, the magnitudes of the fold changes were larger for transcripts that were more abundant at peak parasitaemia compared to those that were more abundant in ascending parasitaemia, for which only smaller (<2-fold) significant changes were observed. The 804 transcripts were further sorted to exclude transcripts where the magnitude of change was less than 2-fold, and this generated a list of 372 genes. When transcripts for which no protein product was annotated were excluded, 322 genes remained. This list was further reduced to 170 genes when transcripts described as VSG were excluded; of these 170 genes, 118 were ≥3-fold more abundant in peak relative to ascending parasitaemia, and 65 were ≥4-fold more abundant in peak relative to ascending parasitaemia (S1 Table).

### Transcripts elevated at peak parasitaemia

The 804 transcripts that showed significant changes between peak and ascending parasitaemia were grouped by the description of their protein product. The category that was most represented for transcripts more abundant at peak parasitaemia was ‘VSG’, followed by ‘hypothetical protein’ (S2a Fig). The predominant categories for transcripts with reduced abundance at peak parasitaemia were ‘hypothetical protein’, followed by ‘other’ (S2b Fig), this including transcripts predicted to be associated with proliferation (e.g. paraflagellar rod protein, DNA polymerase catalytic subunits, and a putative cell division protein kinase) and three putative amino acid transporters.

To assist with functional assignment for those transcripts differentially regulated between ascending and peak parasitaemia, a BLASTP search with the encoded *T*. *congolense* protein sequences was carried out against *T*. *b*. *brucei* 927 proteins and the output was interrogated for genes known to be linked to differentiation. Searching for the stumpy specific marker PAD1, revealed a putative orthologue, TcIL3000.0.18180 but this was not significantly more abundant in peak relative to ascending parasitaemia. However, this molecule was also related to at least 2 other *T*. *congolense* predicted proteins (TcIL3000.7.5000, TcIL3000.0.02160) similar to the PAD gene family in *T*. *brucei*, comprising members of cell surface phylome family 58 [11]. Assignment of reads to differentially expressed members of the family might suppress any obvious elevation in peak parasitaemia samples. Moreover, TcIL3000.0.18180 was itself quite abundant in ascending phase *T*. *congolense*, which may reflect an early elevation of its expression prior to peak parasitaemia similar to the expression of PAD1 mRNA (but not protein) in intermediate form *T*. *brucei* [33].

To interrogate those transcripts most upregulated (i.e. at least 4-fold) at peak parasitaemia in *T*. *congolense*, *T*. *brucei* orthologues of the gene cohort (where available) were analysed for a loss-of-fitness phenotype following RNAi at any *T*. *brucei* life cycle stage, but particularly differentiation[34], and also cell surface phylome family membership[11]. Seven genes in the cohort of 65 elevated transcripts generated a defect in differentiation as determined by genome-wide RNAi fitness screening. Of these, a putative UDP-Gal or UDP-GlcNAc-dependent glycosyltransferase (orthologue Tb927.8.7140) exhibited a fitness deficit restricted only to differentiation, as opposed to bloodstream or procyclic form growth. More strikingly, however, of the strongly elevated transcripts, 46 transcripts (or the predicted *T*. *brucei* orthologue) were annotated as encoding cell surface phylome members (35 transcripts when those belonging to surface family groups described as VSG were excluded). Of these remaining 46 transcripts, 29 transcripts belong to orthogroup OG5_127658 [31], an orthogroup being defined as genes derived from a single gene in the last common ancestor of the species groups. This orthogroup incorporates proteins of cell surface phylome group 15, which is shared between *T*. *brucei* and *T*. *congolense* but whose membership is considerably expanded in *T*. *congolense*. This family contains ESAG6 and 7 proteins associated with transferrin uptake in *T*. *brucei* (see later).

**Fig 3:**
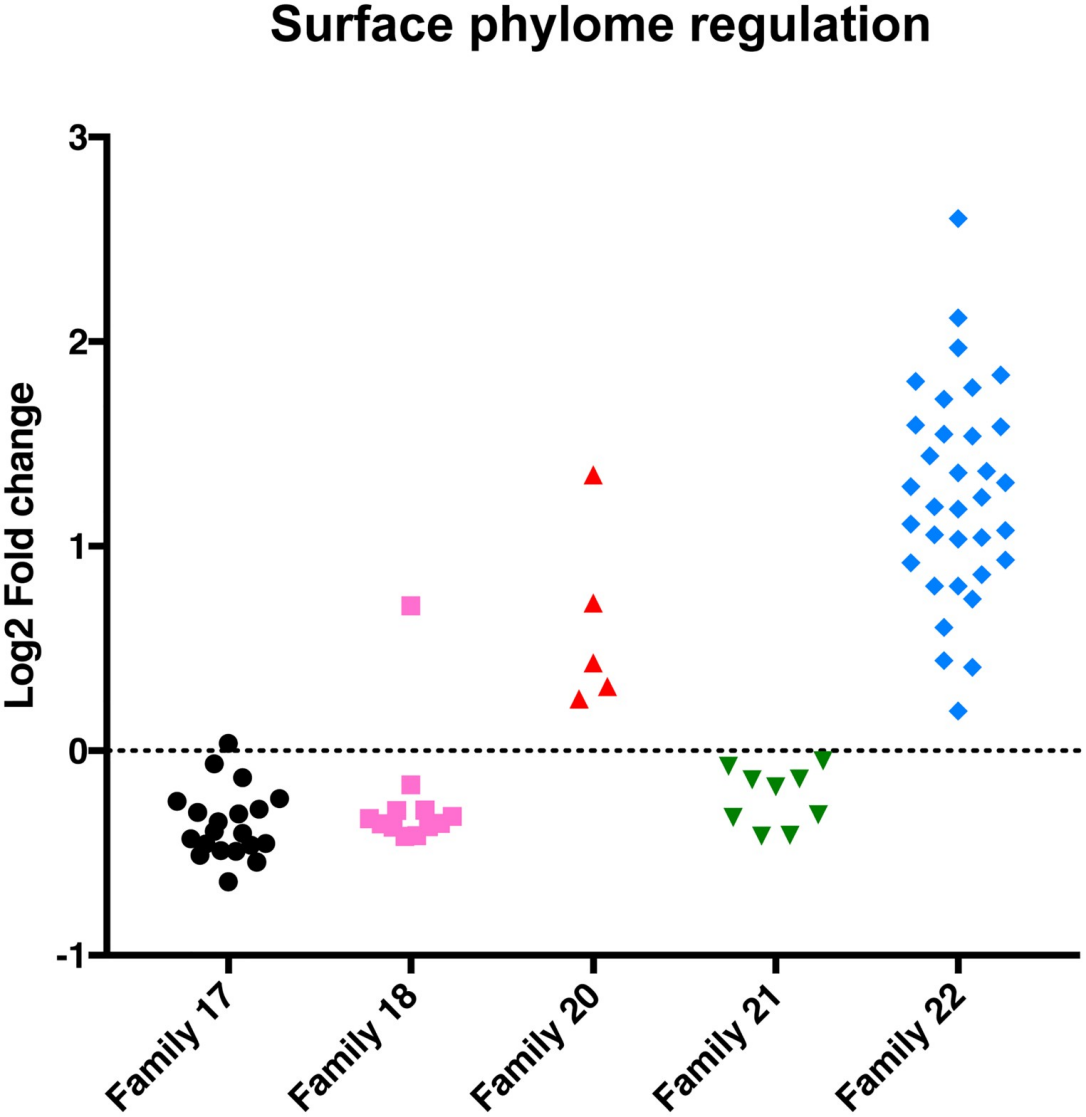
*T*. *congolense*-specific cell surface phylome regulation. Log2 fold change (FC) for *T*. *congolense*-specific cell surface phylome families. Members of Family 20 and Family 22 are upregulated at peak parasitaemia whereas other cell surface phylome families are not upregulated. Note that the fold change in expression levels of all family members are shown irrespective of their statistical significance.

The enrichment of cell surface phylome family 15 in *T*. *congolense* prompted us to explore the developmental regulation of other cell surface phylome members, and particularly those restricted only to *T*. *congolense* (Fig 3). Analysis of the expression profile of members of these *T*. *congolense* species-specific family groups (families 17, 18, 20, 21, 22) demonstrated that members of families 17, 18 and 21 were downregulated in *T*. *congolense* at peak parasitaemia, albeit to different levels and with varying levels of statistical support. In contrast, every identified representative of Family 22 was elevated at peak parasitaemia (with a range between logFC 0.19–2.60) (Fig 3), this also being observed when gene-specific reads were considered (S3 Fig). Family 22 comprises a VSG-related protein family of 175–186 amino acids whose open reading frame is always closely adjacent to, or within the 3’UTR, of a VSG gene. The predicted protein sequence of Family 22 members is not related to other known proteins or protein features and could also represent a ncRNA. Similar to Family 22, all Family 20 members were also upregulated at peak parasitaemia, although the overall level of regulation was more modest. Interestingly, Family 12 members, which comprise the *T*. *brucei* procyclins and the *T*. *congolense* related molecules (TcIL3000.0.53640; TcIL3000.0.02860; procyclin like) were not upregulated in the *T*. *congolense* peak parasitaemia samples; nor were GARP or CESP family members (cell surface phylome family 50).

### Predicted protein domains in hypothetical proteins elevated at peak parasitaemia

Among the *T*. *congolense* transcripts at least 4-fold more abundant at peak relative to ascending parasitaemia (adj p<0.05) there were 23 proteins described as ‘hypothetical’. The amino acid sequences for these 23 hypothetical proteins were searched using the InterPro search tool to identify potential domains of interest (S2 Table). Seven of these proteins were predicted to have one transmembrane domain by the Phobius tool[35], and 3 of these predictions were also supported by TMHMM[36]. Further, 3 of the hypothetical proteins were predicted to have more than one transmembrane domain by Phobius, and in some cases this was supported by predictions by TMHMM. None of these hypothetical proteins were described as part of the *Trypanosoma* cell surface phylome[11], and so they may localize to internal membranes.

**Fig 4:**
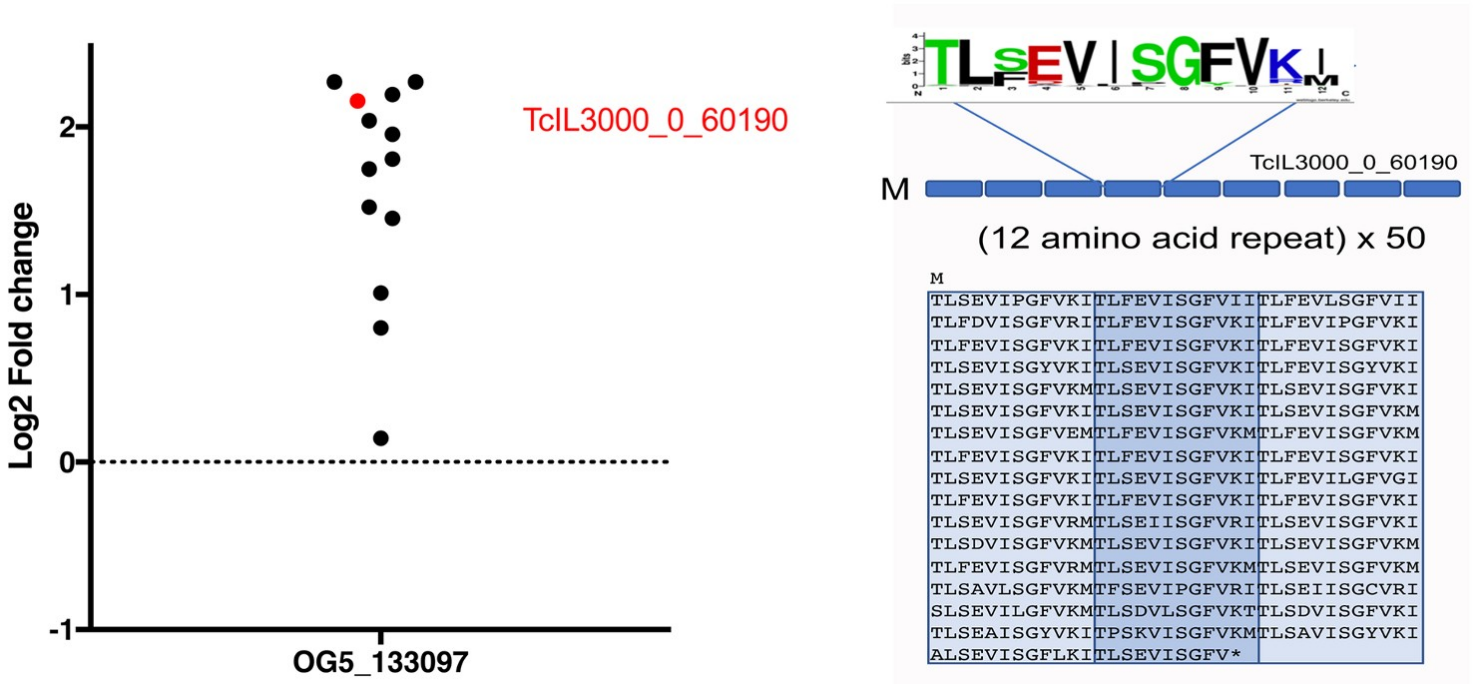
Regulatory profile of Orthogroup OG5_133097 in *T*. *congolense*. Members of orthogroup OG5_133097 encompass predicted proteins comprised of 12 amino acid repeats. Many members of this *T*. *congolense* enriched orthogroup are upregulated at peak parasitaemia, with TcIL3000_0_60190 being 4.5 fold up regulated at peak parasitaemia (adj p = 0.033). This predicted protein comprises 50 copies of an imperfect 12 amino acid repeat.

Among the enriched transcripts encoding hypothetical proteins was also TcIL3000_0_60190 (logFC 2.156), annotated as a dicer-like protein. In fact, this molecule forms a member of orthogroup OG5_133097 which incorporates a family of proteins largely comprised of imperfect 12 amino acid repeats. There are 25 members of this family in *T*. *congolense*, out of 33 in the orthogroup, no other members being found in other kinetoplastids. The protein encoded by TcIL3000_0_60190 contains a predicted signal peptide, N-terminal transmembrane domains (2) and BLASTP similarity to adhesins, adherence factor and cell agglutination proteins (S4 Fig). Interestingly, all members of this orthogroup identified in our datasets exhibited transcript elevation at peak parasitaemia, though many fell below the significance threshold (Fig 4).

### Comparison of *T*. *congolense* transcriptomes with *T*. *brucei* slender and stumpy RNA profiles

**Fig 5:**
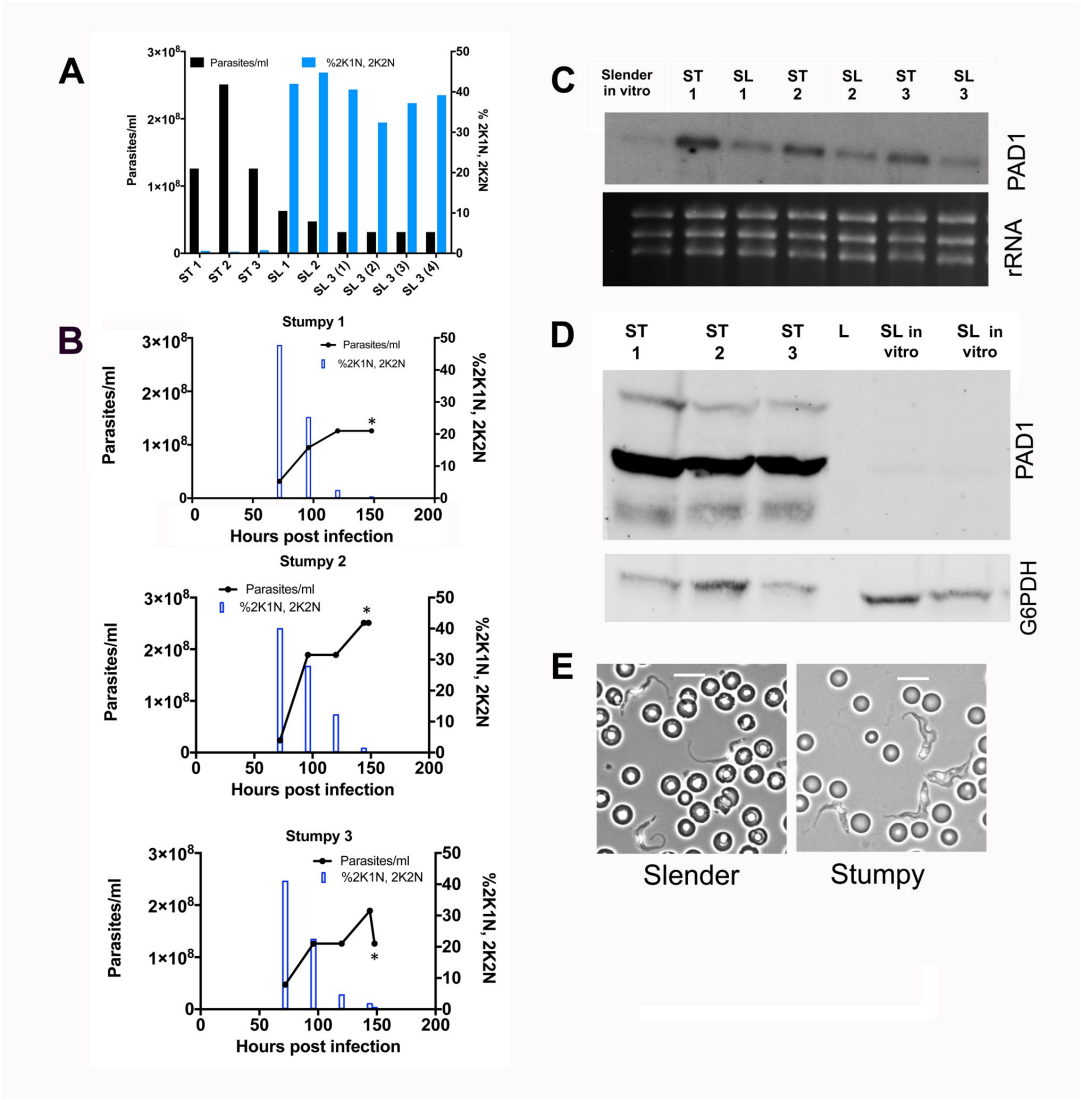
Biological characterisation of *T*. *brucei* material for expression analysis. (A.) Parasitaemia (black bars) and proliferation status (blue bars) for each sample used for transcriptome analysis of *T*. *brucei* slender and stumpy forms. The proportion of parasites presenting 2 kinetoplast, 1 nucleus (2K1N), or 2 kinetoplast 2 nuclei (2K2N) configurations was assessed in 500 cells at each time point, providing a measure of proliferating cells in the population. (B.) The progression of the parasitaemia and proliferation status throughout the parasitaemia of each of the infections used to prepare stumpy form mRNA, highlighting the cell cycle arrest of parasites as they develop toward stumpy forms. Samples were harvested for RNA on day 6 (indicated by an asterisk) (C.) Northern blot of each mRNA sample used for transcriptome analysis hybridised to a riboprobe for the stumpy specific marker, PAD1. Stumpy samples express more PAD1 mRNA than slender samples. (D.) PAD1 protein expression for the stumpy samples used for transcriptome analysis. *In vitro* cultured slender samples provide a negative control. Loading is indicated by detection of G6PDH (E.) Morphological analysis of the slender form samples (left hand side) or stumpy forms (right hand side) in blood. Scale bar represents 10μm.

To act as comparator for the *T*. *congolense* ascending and peak parasitaemia RNA profiles, transcriptome datasets were generated from *T*. *brucei* slender and stumpy form parasites isolated from mouse infections (Fig 5). Thus, parasites from infections with *T*. *b*. *brucei* EATRO 1125 AnTat 1.1 90.13 were collected at either ascending parasitaemia when parasites were of predominantly slender morphology, or at peak parasitaemia when parasites were of stumpy morphology. Triplicates were prepared for each developmental form, with the stumpy RNA samples and two of the slender RNA samples each derived from an individual *T*. *b*. *brucei* EATRO 1125 AnTat 1.1 90.13 infection. The third slender RNA sample was derived from a pool of 4 infections to generate sufficient material for RNA-Seq analysis. Matching the analysis in *T*. *congolense* infections, the cell cycle status of the parasite isolates was also analysed, with proliferative slender cells expected to be enriched for the 2K1N/2K2N configuration with respect to G1/G0-arrested stumpy forms (the parasitaemias and associated cell cycle data for each sample are shown in S1 Fig). Fig 5A and 5B confirms that the parasites used to prepare the stumpy RNA samples were enriched in 1K1N, whereas those parasites used to prepare slender RNA samples were proliferative with a greater proportion of cells that had either a 2K1N or 2K2N configuration (Fig 5A and 5B). Fig 5C shows the isolated total RNA analysed by Northern blotting using a probe targeting PAD1, confirming that the levels of the transcript were higher in the stumpy RNA samples than in the slender RNA samples (Fig 5C), although some transcript was detected perhaps reflecting the beginning of development to intermediate forms in the isolated slender cells (intermediate forms express as much PAD1 mRNA as stumpy forms;[37]). Further validation of the developmental status was obtained by western blotting using a PAD1 specific antibody (Fig 5D), which confirmed that the levels of PAD1 protein expression were considerably higher in the protein samples from the parasites used to isolate stumpy RNA than in control protein samples from slender *T*. *b*. *brucei* EATRO 1125 AnTat 1.1 90.13 parasites cultured *in vitro*. An equivalent analysis of the material used to generate slender RNA for RNA-Seq analysis could not be carried out due to the low parasitaemia for these samples, but morphological analysis confirmed the slender and stumpy morphotypes for parasites from which each slender and stumpy RNA sample was derived. (Fig 5E).

### Alignment to the *T*. *brucei* genome and analysis of differential expression

**Fig 6:**
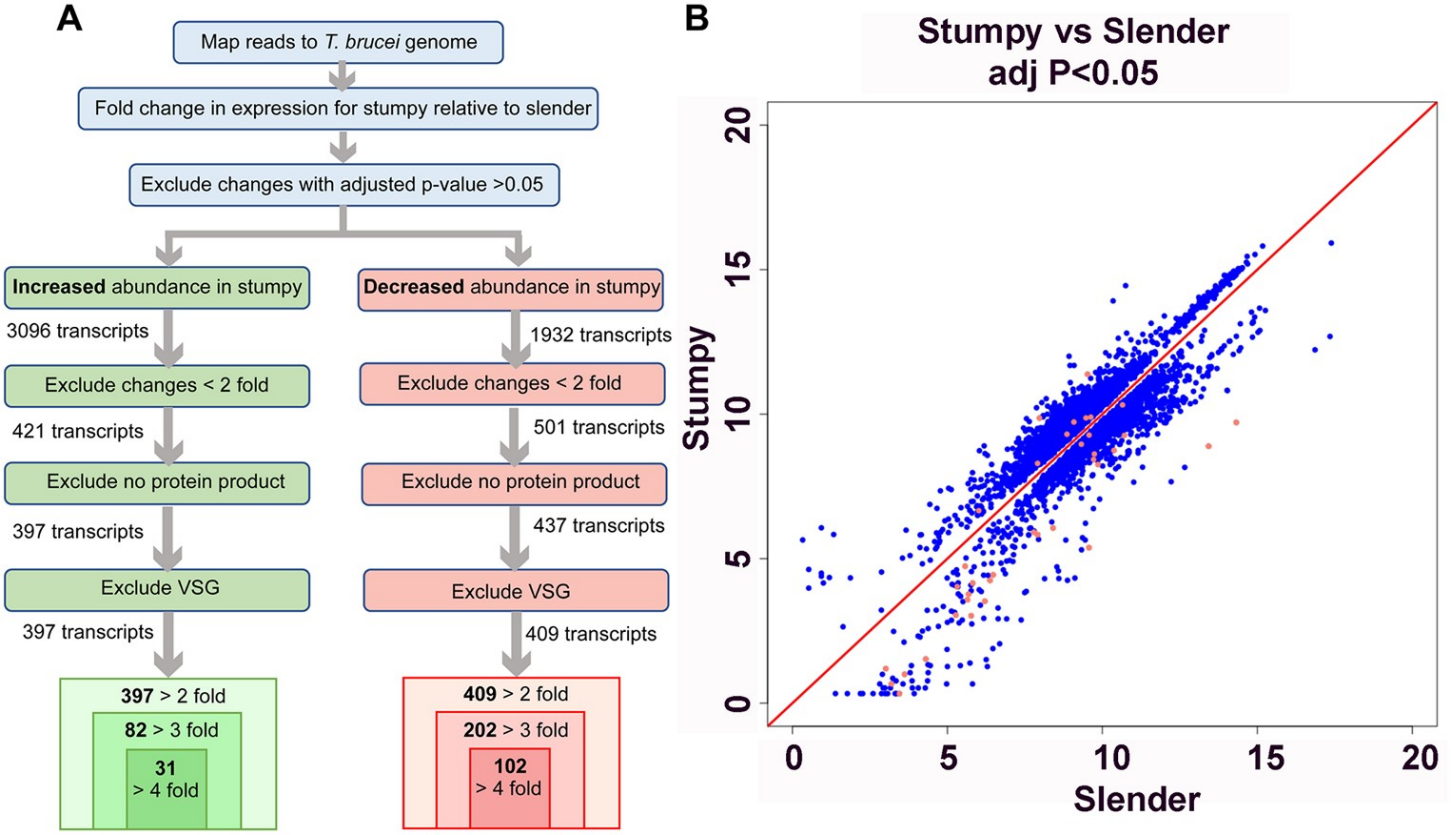
Expression differences between slender and stumpy form *T*. *brucei*. (A.)Strategy for the comparison of mRNA expression between slender and stumpy form *T*. *brucei*. (B.)Scatterplot showing transcripts that show significant (adjusted p value <0.05) expression difference between slender and stumpy samples. Transcripts annotated as VSG are coloured red.

Matching the analysis of *T*. *congolense* ascending and peak parasitaemia samples, slender and stumpy RNA-Seq reads were processed to generate Log2FC values comparing developmental forms. In total, 5028 genes demonstrated significant fold changes in abundance (adj. P-value <0.05) (Fig 6A and 6B). Of these, 3096 transcripts were increased in stumpy relative to slender parasites, and 1932 transcripts were decreased in stumpy relative to slender parasites (S3 Table). When changes of less than 2-fold magnitude were excluded, 421 transcripts were at least 2-fold more abundant in stumpy relative to slender parasites, and 501 transcripts were at least 2-fold less abundant in stumpy relative to slender parasites. This contrasts with the absence of transcripts downregulated at least 2-fold in peak versus ascending parasitaemia *T*. *congolense* samples.

Transcripts were further sorted to exclude those annotated as VSG or with no protein product assigned. In comparison to *T*. *congolense*, relatively few regulated transcripts were annotated as VSG and, of those transcripts regulated at least 2-fold that were described as VSG, all were decreased in abundance in stumpy relative to slender parasites.

The 3096 transcripts significantly increased, or 1932 transcripts significantly decreased, in stumpy relative to slender form parasites, were sorted by their protein product description (S5 Fig). The most common category of differing transcript abundance between stumpy and slender forms was ‘hypothetical proteins’; there are 3460 genes annotated to encode ‘hypothetical proteins’ out of 9729 protein coding genes in the genome overall. Other well-represented categories included protein kinases, phosphatases, and RNA-binding proteins. An increase in abundance of transcripts encoding transporter proteins, and particularly amino acid transporters, was seen whereas glucose transporters were downregulated. There was also an upregulation of procyclin and BARP surface protein transcripts consistent with the preparation for transmission. Additionally, consistent with changing metabolic requirements on transmission, transcripts described as mitochondrial carrier proteins, or as NADH-ubiquinone oxidoreductase (part of the mitochondrial electron transfer chain in procyclic forms, [38]), were also increased in abundance in the stumpy form, as would be expected for this life cycle stage with a more elaborated mitochondrion than slender forms [14].

As previously noted, the ESAG9 family were significantly increased in abundance in stumpy forms [39, 40]. Similarly, the protein-associated with differentiation 1 (PAD1) transcript, alongside those of PAD2 and 3, were found to be of increased abundance in stumpy forms. However, the fold change in PAD1 was not as high as PAD2, probably due to some PAD1 elevation due to its early expression in intermediate forms present in the isolated ‘Slender’ material used. Also matching expectation, increased abundance of the PTP1-interacting protein, TbPIP39, transcript was observed in the stumpy-enriched data set [41]. In a study of early proteomic changes made during commitment to differentiation to the procyclic form [42] TbPIP39 protein abundance increased 3 hours after induction of differentiation, alongside a trans-sialidase and eukaryotic initiation factors. The increased abundance of these transcripts in stumpy forms is likely a necessary preadaptation for the rapid proteomic changes required after transmission.

With respect to transcripts downregulated in stumpy forms, these included flagellar protein, histone, and beta-tubulin transcripts, consistent with the growth-arrested status of the quiescent transmission stage. Additionally, transcripts for the haptoglobin-haemoglobin receptor (Tb927.6.440) were reduced in abundance in the stumpy form as expected[43].

In combination, these data confirmed that the slender and stumpy RNA-Seq samples corresponded to the known characteristics of these developmental forms, validating them as a suitable comparator for *T*. *congolense* ascending and peak parasitaemia forms.

### Comparison of *T*. *brucei and T*. *congolense* density-dependent differentiation

**Fig 7:**
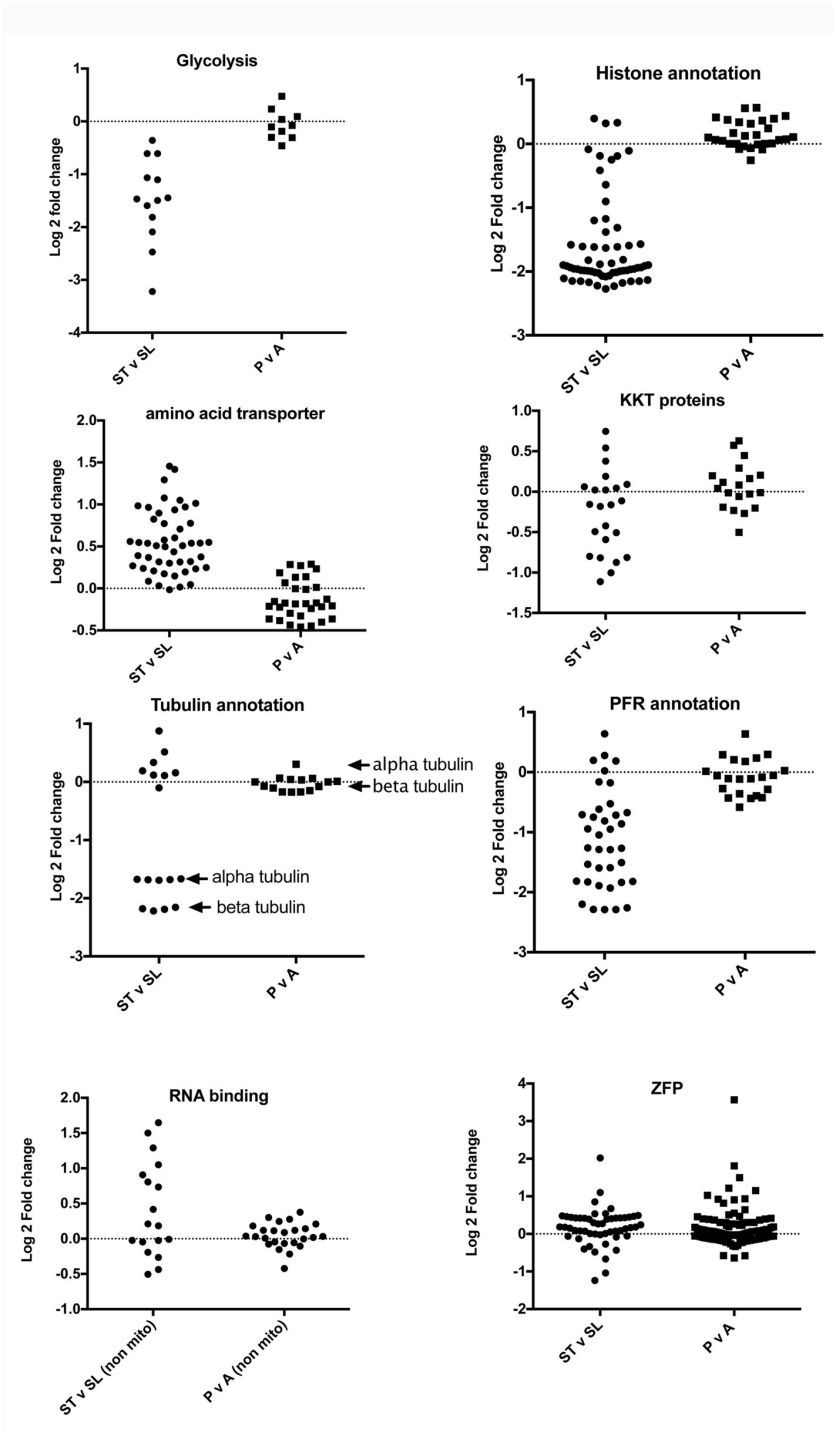
Differential expression of components of different biological processes in *T*. *congolense* and *T*. *brucei*. Log2 fold change of transcripts associated with different functional processes in peak versus ascending parasitaemia *T*. *congolense*, or stumpy versus slender *T*. *brucei*. For glycolysis, the relevant pathway enzymes were analysed (and their orthologues in *T*. *congolense*). For the other comparisons, annotation-based criteria (‘histone’, ‘amino acid transporter’, ‘KKT protein’, ‘tubulin’, ‘paraflagellar rod’, ‘RNA binding’ or ‘zinc finger protein’) were used to analyse each functional class. In the case of ‘histones’ this also included histone modifying enzymes; for the ‘RNA binding’ group this included pumillio class proteins as well as non-mitochondrial RNA binding proteins.

To compare regulated processes between peak parasitaemia forms from *T*. *brucei* and *T*. *congolense*, we specifically analysed the expression of genes involved in several cellular functions that are linked to developmental regulation and cellular quiescence in *T*. *brucei*. Namely, we explored the regulation of transcripts involved in glycolysis, cell proliferation (histone, tubulin, paraflagellar rod transcripts), kinetochore proteins (KKT transcripts; [44]), amino acid transporters and RNA binding proteins (including pumillio proteins, zinc finger proteins and mitochondrial RNA binding proteins). Except glycolysis, where the specific enzymes involved were analysed, in each case protein product descriptions were used as the search term and the expression profile of the cohort plotted with respect to log2 FC between either stumpy and slender forms of *T*. *brucei*, or between peak parasitaemia and ascending parasitaemia *T*. *congolense*. Fig 7 shows the expression profiles exhibited in each transcript cohort, which demonstrated a greater regulatory trend in stumpy forms of *T*. *brucei* than in peak parasitaemia *T*. *congolense* with respect to their proliferating ascending parasitaemia counterparts. Thus, most enzymes involved in glycolysis were down regulated in stumpy forms consistent with their quiescence, whereas no change between peak and ascending parasitaemia was seen in *T*. *congolense*. Similarly, mRNA markers of cell proliferation, such as histone, paraflagellar rod protein and alpha and beta tubulin transcripts were strongly downregulated in stumpy forms but these were largely unchanged in the *T*. *congolense* peak parasitaemia samples. The recently identified and divergent kinetochore proteins also showed distinct regulation in stumpy forms compared to slender forms, and this was less pronounced in *T*. *congolense*. Contrasting with the down regulation of the aforementioned transcripts in quiescent stumpy forms, transcripts annotated as amino acid transporters were predominantly upregulated whereas in *T*. *congolense* no general increase was observed. The regulatory pumillio class of RNA binding protein mRNAs were also upregulated in stumpy forms, perhaps associated with their role in translational regulation, as were those annotated ‘mitochondrial RNA binding protein’, however an equivalent regulation was not observed in *T*. *congolense*. Zinc finger proteins, known to be important in several development events in *T*. *brucei* were not consistently upregulated as a group in either *T*. *brucei* stumpy forms or *T*. *congolense* peak samples, although two transcripts were highly elevated: TcIL3000_0_11070 in *T*. *congolense* (Log2 FC 3.56; adj p value = 0.057) and Tb927.5.810 (ZC3H11) in *T*. *brucei* (Log2 FC 2.01; adj p value = 0.001).

### Comparison of regulated orthogroups in *T*. *brucei* and *T*. *congolense*

**Fig 8:**
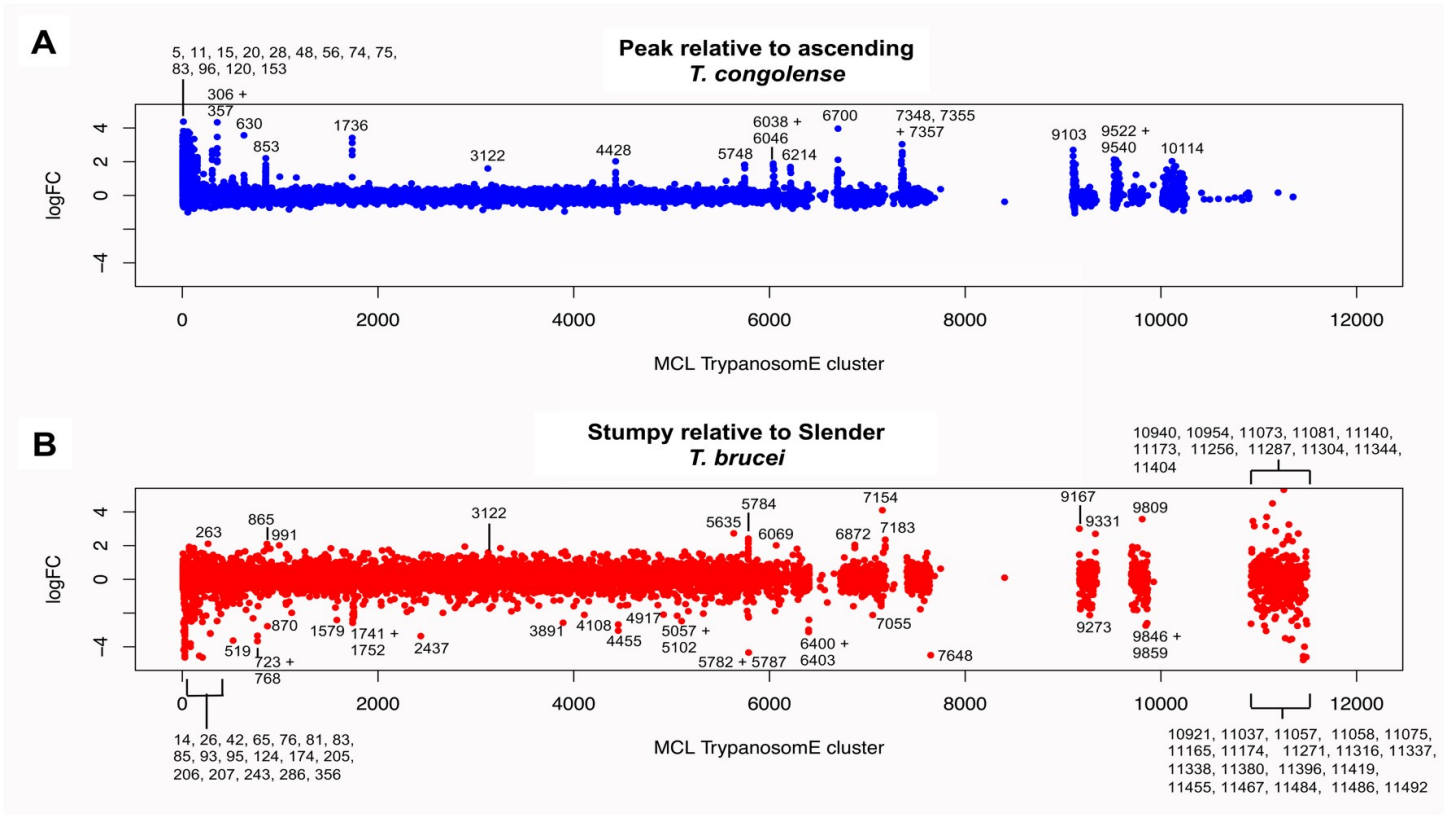
Differential orthogroup expression in *T*. *congolense* and *T*. *brucei*. (A.) Log2 fold change between peak and ascending parasitaemia *T*. *congolense* of all transcripts aligned according to their MCL TrypanosomE orthologous grouping. Groups that show Log2FC of 2 or more in at least one member are annotated. (B.) Log2 fold change between stumpy and slender form *T*. *brucei* of all transcripts aligned according to their MCL TrypanosomE orthologous grouping. Groups that show Log2FC of 2 or more in at least one member are annotated.

To expand the comparison of the *T*. *brucei* and *T*. *congolense* data sets based on biological function, the transcripts identified by RNA-Seq were placed into orthologue groups based on OrthoMCL [31]and regulation of orthogroup members was compared between species. When the Log2FC values for *T*. *congolense* ‘peak relative to ascending’ parasitaemia were plotted against orthologue cluster, certain clusters had multiple members with pronounced increases in abundance at peak parasitaemia. These orthologous groups did not display the same trend in the *T*. *brucei* ‘stumpy relative to slender’ plot (Fig 8a; S4 Table) and many of the *T*. *congolense* peak parasitaemia-enriched orthologous groups that contained hypothetical proteins were *T*. *congolense* specific or enriched (S5 Table). This included members of the aforementioned FAM22 cell surface family[11]. Also upregulated, as examples, were orthogroups 1736 (GRESAG4), 4428 (ESAG3), 306 (cysteine rich acidic membrane proteins; comprising hexamer repeat sequences), 6700 (SecA-DEAD-like domain containing protein), and 9522 (glycosyl transferase proteins). Similarly, the analysis for ‘stumpy versus slender’ enriched *T*. *brucei* transcripts identified the *T*. *brucei* specific ESAG9 orthogroup (Cell surface phylome family 2), plus procyclin (Cell surface phylome family 12) and hypothetical protein containing orthogroups. To directly compare the regulation of orthologue clusters shared between the two trypanosome species, the mean log fold change (Log2FC) from ascending to peak *T*. *congolense* parasitaemia was calculated for each orthogroup and compared with the same analysis in *T*. *brucei*. Focusing on those orthogroups for which the mean *T*. *congolense* fold change in abundance was greater than two, in most cases the mean change in abundance for stumpy relative to slender forms was also positive for these orthologue groups, but in only one case (cluster 3122) was the mean change in abundance of comparable magnitude between the *T*. *brucei* and *T*. *congolense* data sets (Fig 8). Cluster 3122 members are described as conserved hypothetical proteins, and RNAi-mediated knock down of the *T*. *brucei* orthologue generates a loss-of-fitness on differentiation from bloodstream to procyclic forms[34]. An InterPro search using the *T*. *congolense* and *T*. *brucei* sequences from cluster 3122 did not reveal any predicted domains.

**Fig 9:**
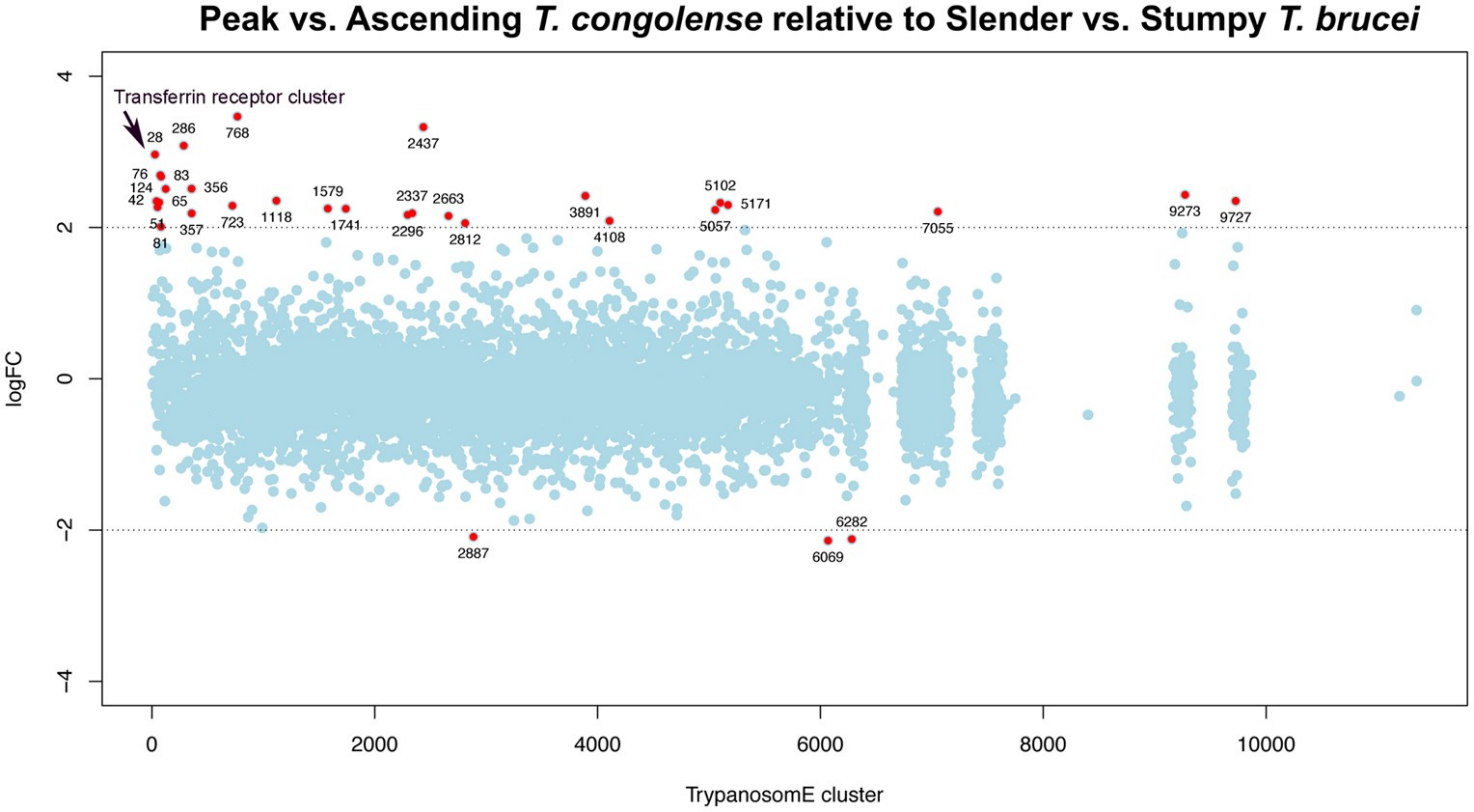
Comparative orthogroup regulation in *T*. *congolense* and *T*. *brucei* differences between mean Log2FC values for orthologous group expression in peak versus ascending parasitaemia *T*. *congolense* and stumpy versus slender *T*. *brucei*. A positive LogFC value indicates an orthologous group for which the fold change was greater for *T*. *congolense* ascending to peak parasitaemia than for *T*. *brucei* slender to stumpy differentiation, with a negative LogFC value indicating that the fold change was greater in the *T*. *brucei* samples. MCL TrypanosomE orthogroups showing at least 4-fold difference between species are shown in red.

**Fig 10:**
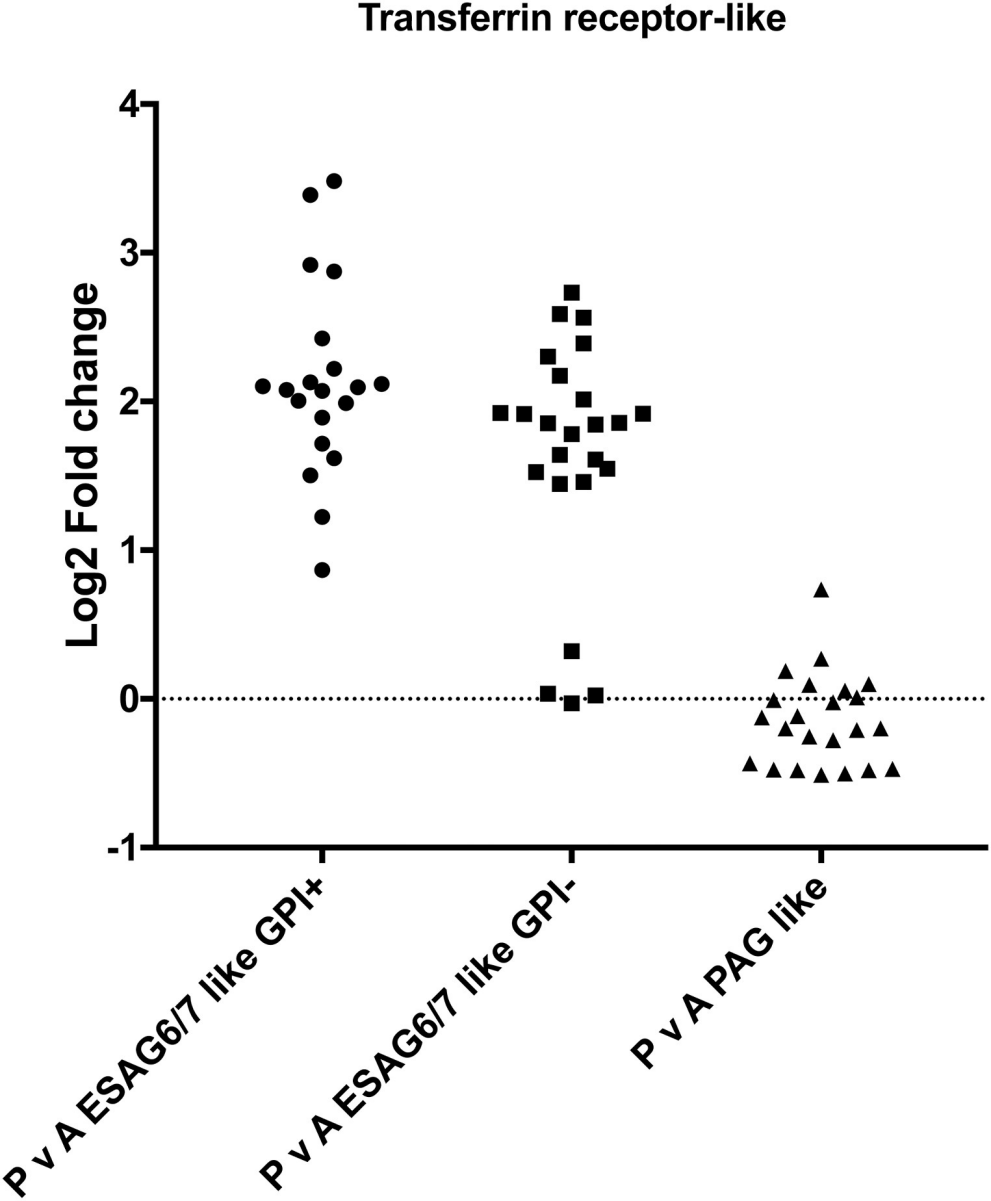
Transferrin family regulation in *T*. *congolense* in ascending or peak parasitaemia. Log2 fold change between peak and ascending parasitaemia of *T*. *congolense* members of the cell surface phylome family 15 (ESAG6-like transferrin binding, with or without a predicted GPI anchor) and family 14 (PAG-like transferrin binding).

Finally, for the orthologue clusters shared between *T*. *brucei* and *T*. *congolense*, we plotted the differences between the mean Log2FC values for *T*. *congolense* ‘peak versus ascending’ data and *T*. *brucei* ‘stumpy versus slender’ data (Fig 9). Clusters with the largest differences between *T*. *congolense* and *T*. *brucei* are labelled with the cluster ID and summarised in S6 Table. Beyond orthogroups involved in cell proliferation (histones, PFR, etc., as highlighted earlier) the most pronounced differentially-regulated orthogroup between *T*. *brucei* and *T*. *congolense* was the ‘Transferrin receptor (TFR)- like’ orthogroup highlighted earlier as significantly enriched in peak parasitaemia *T*. *congolense*. This orthogroup has many more members in the *T*. *congolense* genome than in *T*. *brucei* and includes ESAG6 like (Surface Phylome Family 15; 45 genes) and PAG like (Surface Phylome Family 14; 31 genes) groups. Analysis of the expression of each type, revealed that it is specifically the ESAG6 TFR family that is upregulated as a group, and that the PAG like TFR group are largely unregulated between ascending and peak parasitaemia parasites (Fig 10, S3 Fig). Since the transferrin receptor of *T*. *brucei* is a heterodimer, with one of the components possessing a GPI anchor modification, we predicted the presence or absence of this modification on those transcripts upregulated in peak parasitaemia *T*. *congolense* using both the big-PI [45] and PredGPI [46] predictive algorithms. This revealed that approximately 45% of those transcripts encoding TFR were predicted to have a GPI anchor modification whereas 55% were not. This predicts that at peak parasitaemia transcripts encoding a functional heterodimer were upregulated, rather than one or other constituent components of the functional receptor.

## Discussion

The developmental cycles of different African trypanosome species differ dramatically in their tsetse fly vector, with *T*. *brucei* undergoing maturation in the salivary glands after proliferation in the midgut, whereas *T*. *congolense* matures in the proboscis or cibarium of the fly. Both species however, initially occupy the tsetse midgut and it would be expected that similar adaptations for transmission may be shared by these different parasite species. A key adaptation for transmission occurs in the bloodstream of mammalian hosts, whereby *T*. *brucei* generates an alternative developmental stage, the stumpy form, that is less sensitive to protease exposure or pH fluctuations, and which undergoes efficient differentiation to tsetse midgut procyclic forms *in vivo* and in culture. *Trypanosoma congolense*, by contrast, does not develop a morphologically-distinct stumpy form but has been shown recently to undergo cell-cycle arrest when at peak parasitaemia, reminiscent of the cell quiescence exhibited by mature stumpy forms. *T*. *congolense* also conserves the machinery identified in *T*. *brucei* as needed for density-dependent differentiation in the bloodstream [28]. In the work described here we sought to compare the gene expression changes between proliferating and arrested forms of each parasite species (slender/stumpy for *T*. *brucei*; ascending/peak parasitaemia for *T*. *congolense*) to identify common or distinct gene expression patterns in response to the density of each species in the bloodstream of their mammalian host. With respect to *T*. *brucei*, our *ex vivo* datasets do not compare well with those of a recent study using *in vitro* grown parasites when considering transcripts that show altered expression between slender and stumpy forms. This may reflect differences between *ex vivo* and *in vitro* derived parasites, mRNA isolation methods and/or inter-laboratory variation [47].

Overall our analyses highlight that *T*. *brucei* undergoes more extreme adaptation, with the downregulation of transcripts linked to cellular proliferation and metabolism and the upregulation of molecules required in the tsetse midgut after transmission. Although *T*. *congolense* also exhibits reduced proliferation at elevated parasite density, these parasites show much less evidence of progression to cellular quiescence, matching their absence of morphological transformation. Nonetheless, the parasites do not simply exhibit reduced proliferation; rather they significantly up-regulate transcripts encoding several specific surface protein families upon their development at peak parasitaemia. This indicates that, although monomorphic, these parasites exhibit development within the bloodstream and that this is associated with the expression of specific surface molecules that may promote infection chronicity or assist parasite survival in the mammalian host, or during their transmission to tsetse flies.

Analysis of the cell surface phylome of different trypanosome species highlights the existence of 5 non-VSG protein families that are specific to *T*. *congolense*. In addition, there are four protein families shared with *T*. *brucei* (but not *T*. *vivax*) of which two, Family 14 and Family 15 are expanded in *T*. *congolense*. Of the *T*. *congolense* specific surface protein families, Families 17, 18 and 21 did not change in expression as a cohort between ascending and peak parasitaemia, but Family 20 and particularly Family 22 members were upregulated during the development to the peak parasitaemia form, accounting for both reads assigned randomly between members for specific gene families and uniquely mapped reads (S3 Fig). Similarly, of those families shared with *T*. *brucei*, the expanded Family 15 surface proteins were upregulated, contrasting with the Family 14 cohort despite both Families being annotated as transferrin binding. Thus, elevated expression of Family 15, 20 and 22 members defines the peak parasitaemia forms of *T*. *congolense*.

Of the upregulated surface protein families, Family 20 represents a family of small (~107 amino acid) proteins that show no homology to other proteins and are of unknown function. Family 22, in contrast, represents a much larger gene family with over 100 representatives, these encoding proteins of approximately 180 amino acids. The genes occupy an interesting genomic location, being found directly downstream of sub-telomeric VSG gene sequences and potentially positioned within the 3’UTR of these genes. As with Family 20 members, however, the predicted protein sequences do not provide any indication of the likely function of the family, although a subset do contain a predicted signal peptide, suggesting surface expression of at least some members. In contrast, Family 15 transcripts encode ESAG6 related transferrin receptor proteins. The Transferrin receptor ESAG6/7 is encoded as an integral component of the *T*. *brucei* VSG gene expression site and the proteins form a heterodimer to allow iron uptake. The trypanosome transferrin receptor proteins are predicted to be derived from VSG sequence, and this has diverged in *T*. *congolense* to an ESAG6 related family (represented by Family 15) and a further family related to the procyclin associated genes (represented by Family 14). Of these two families, only Family 15 was upregulated in *T*. *congolense* peak parasitaemia parasites, with isoforms predicted to be modified by GPI addition and those without this modification upregulated, suggesting regulation of functional heterodimers. Their upregulation in *T*. *congolense* at peak parasitaemia perhaps relates to an increased need to scavenge transferrin at high parasite numbers, or when stressed at peak parasitaemia, although the peak parasitaemia levels in *T*. *congolense* were equivalent to the parasite levels for the isolated *T*. *brucei* stumpy samples. Sensitivity to iron depletion may be relevant for *T*. *congolense* in chronic livestock infections where trypanosome infection can be characterised by anaemia and limited iron availability as an anti-pathogen defence mechanism [48].

Beyond the described cell surface phylome members, other transcripts upregulated at peak parasitaemia in *T*. *congolense* were of potential interest. These included representatives of orthogroup OG5_133097, all members of which were upregulated, although only one was upregulated more than 4 fold (adj p<0.05). These transcripts are predicted to encode a family of proteins with imperfect 12 amino acid repeats comprising the majority of the protein sequence. Although functionally uncharacterised, BLAST searches highlighted similarity to proteins involved in cell adhesion or agglutination. This is intriguing with respect to *T*. *congolense*, which is characterised by binding to culture flasks when grown as bloodstream forms. Indeed, the parasites adhere in the vasculature and in the proboscis during life in the blood or tsetse fly. Hence the expression of these proteins at peak parasitaemia may promote transmission by causing the parasites to accumulate, for example, in the skin. Alternatively, these proteins could contribute to virulence in the mammalian host through enhanced adhesion and perhaps sequestration of parasites. Exploration of these potential functions awaits the development of robust tools for reverse genetic analysis in *T*. *congolense* bloodstream forms.

In comparison to *T*. *brucei* slender to stumpy transitions, the changes in *T*. *congolense* were relatively modest. This reflects the descriptions of these parasites in the literature where a morphological transformation is not obvious and the mitochondrial activity of the parasite is already well developed in the bloodstream, unlike in *T*. *brucei* where the slender form has a much simpler and underdeveloped mitochondrion than in stumpy or tsetse fly forms[14]. Recently, an analysis by flow cytometry of the parasites used in this study, *T*. *congolense* IL3000, confirmed the absence of a clear morphological transition at peak parasitaemia despite their cell cycle arrest [28]. Whilst this could reflect the particular stock used, which has been subject to long term passage in rodents, other more recent field isolates also show a lack of clear morphological transformation, suggesting that monomorphism is a general feature of these parasites, rather than a laboratory adaptation. In the absence of morphological transformation, the question of whether peak parasitaemia *T*. *congolense* represents a distinct developmental form, like the stumpy form, becomes relevant. A number of lines of evidence suggest that there is developmental adaptation. Firstly, the parasites conserve the signalling pathway for quorum sensing exploited in *T*. *brucei* and at least one component is able to functionally complement a *T*. *brucei* null mutant to restore stumpy formation [28]. Secondly, although the downregulation of transcripts observed with stumpy form quiescence was less obvious in *T*. *congolense*, specific subsets of molecules were strongly upregulated in peak parasitaemia stages, arguing for an active developmental progression rather than a passive cell-cycle arrest only. Finally, *T*. *congolense* isolated at peak parasitaemia may be more able to infect tsetse flies than those during the ascending phase [49] though this has not been the observation in all studies[50]. All of these observations argue for a specific adaptation for transmission in *T*. *congolense* and their lack of morphological adaptation makes it even more important than in *T*. *brucei* that specific molecular markers for that form are identified. The transcriptome datasets generated here provide not only potential molecular signatures of preadaptation but also molecular tools that can help to dissect the transmission biology of *T*. *congolense* and how this compares with the alternative developmental fates of *T*. *brucei* and *T*. *vivax*. This complements recent transcriptome studies derived for tsetse-inhabiting stages for both *T*. *congolense* [51] and *T*. *brucei* [52, 53]. Furthermore, in bacterial systems quorum-sensing regulates expression of virulence factors [54], thus some of the transcripts differentially regulated at low versus high parasite density could represent novel virulence factors in *T*. *congolense* infection.

## Supporting information

S1 Table Differential expression of *T*. *congolense* transcripts between ascending and peak parasitaemia. Functional assignment for the 804 transcripts significantly up or downregulated in the peak parasitaemia *T*. *congolense* transcriptome compared to the ascending parasitaemia transcriptome.
(XLS)

S2 Table Predicted domains in proteins encoded by upregulated transcripts at peak *T*. *congolense* parasitaemia. InterPro search of predicted domains for *T*. *congolense* hypothetical proteins encoded by transcripts at least 4-fold more abundant at peak relative to ascending parasitaemia.
(XLSX)

S3 Table Differential expression of *T*. *brucei* transcripts between slender and stumpy forms. Functional assignment for the 5028 transcripts significantly up (3096 transcripts) or downregulated (1932 transcripts) in the stumpy form *T*. *brucei* transcriptome compared to the slender form transcriptome.
(XLS)

S4 Table Transcripts of increased abundance at *T. congolense* peak parasitaemia do not fall into the same orthologous group as transcripts increased in abundance in *T. brucei* stumpy forms. Orthologous groups with increased expression at *T*. *congolense* peak parasitaemia (Sheet 1), which were distinct from those clusters enriched in (Sheet 2) or reduced in (Sheet 3) *T*. *brucei* stumpy forms.
(XLSX)

S5 Table Trypanosome orthologous groups for which a *T*. *congolense* member is at least 4-fold more abundant at peak parasitaemia than during ascending parasitaemia. The number of *T*. *congolense* transcripts that had significantly altered abundance were listed alongside the total number of *T*. *congolense* transcripts in that cluster. If any genes in the cluster belonged to cell surface phylome families [11] then this was also recorded.
(XLSX)

S6 Table Differences between mean Log_2_FC values for *T*. *congolense* peak vs. ascending data and *T*. *brucei* stumpy vs. slender data. Orthologue clusters (green highlighting) for which the LogFC difference between mean *T*. *congolense* peak versus ascending compared to mean stumpy versus slender expression was >2 and at least one *T*. *congolense* transcript had a significant fold change (adjusted p-value <0.05) in the RNA-seq data. Red Highlighting shows clusters for which the LogFC difference was <-2 and at least one *T*. *b*. *brucei* transcript had a significant fold change (adjusted p-value <0.05) in the RNA-seq data.
(XLSX)

S1 Fig Infection data for *T*. *congolense* and *T*. *brucei* samples. Infection associated data for the material used to analyse *T*. *congolense* ascending and peak parasitaemia parasites and *T*. *brucei* slender and stumpy forms.
(PDF)

S2 Fig Transcripts with significantly changed abundance between ascending and peak *T*. *congolense* parasitaemia that have been grouped by product description. A. Protein description categories for transcripts significantly more abundant at peak parasitaemia than ascending parasitaemia. B. Protein description categories for transcripts with significantly reduced abundance at peak parasitaemia relative to ascending parasitaemia.
(PDF)

S3 Fig Surface phylome regulation comparing randomly assigned and unique reads for different *T*. *congolense* specific surface phylome members. Analysis of the expression of surface phylome members that are unique to *T*. *congolense*. The graph shows assignment to different family members accounting only for unique reads specific to particular members of each family.
(PDF)

S4 Fig BLAST alignment of an upregulated ‘adhesin’ like protein. The image shows the TritrypDB graphical representation of the BLAST hits returned using gene code TcIL3000_0_60190. The graphical representation was derived by running Wu Blast—BlastP against the NR database.
(PDF)

S5 Fig Transcripts with significant differences in abundance between slender and stumpy parasites that have been grouped by product description. A. Transcripts with increased abundance in stumpy relative to slender parasites. B. Transcripts with decreased abundance in stumpy relative to slender parasites.
(PDF)
