# Supplementary material for: A gene expression comparison of Trypanosoma brucei and Trypanosoma congolense in the bloodstream of the mammalian host reveals species-specific adaptations to density-dependent development
Source: PLoS Negl Trop Dis. 2018 Oct 11;12(10):e0006863. doi: 10.1371/journal.pntd.0006863 (PMC6199001; doi:10.1371/journal.pntd.0006863)
Supplement: S1 Fig — Infection associated data for the material used to analyse T. congolense ascending and peak parasitaemia parasites and T. brucei slender and stumpy forms. (PDF) [file pntd.0006863.s007.pdf]

Sample	Species	Pooled or Individual infection?	Collection Day (days post-infection)	Parasitaemia	%2K1N, 2K2N
Slender 1	*T. brucei*	Individual	Day 3	6.31E+07	42
Slender 2	*T. brucei*	Individual	Day 3	4.74E+07	44.8
Slender 3	*T. brucei*	Pooled (x4)	Day 3	3.16E+07	32.4, 37.2, 39.2, 40.6
Stumpy 1	*T. brucei*	Individual	Day 6	1.26E+08	0.6
Stumpy 2	*T. brucei*	Individual	Day 6	2.51E+08	0.4
Stumpy 3	*T. brucei*	Individual	Day 6	1.26E+08	0.8

Sample	Species	Pooled or Individual infection?	Collection Day (days post-infection)	Parasitaemia	%2K1N, 2K2N
Ascend 1	*T. congolense*	Pooled (x3)	Day 5	4.74E+07, 6.31E+07, 2.37E+07	8.8, 10.6, 12.8
Ascend 2	*T. congolense*	Pooled (x3)	Day 5	9.46E+07, 6.31E+07, 4.74E+07	9.4. 16.2, 11.8
Ascend 3	*T. congolense*	Pooled (x2)	Day 5	6.31E+07, 3.16E+07	8.6, 15.4
Peak 1	*T. congolense*	Individual	Day 7	1.89E+08	6.0
Peak 2	*T. congolense*	Individual	Day 7	1.26E+08	5.4
Peak 3	*T. congolense*	Individual	Day 6	2.51E+08	12.4
