# Supplementary material for: A gene expression comparison of Trypanosoma brucei and Trypanosoma congolense in the bloodstream of the mammalian host reveals species-specific adaptations to density-dependent development
Source: PLoS Negl Trop Dis. 2018 Oct 11;12(10):e0006863. doi: 10.1371/journal.pntd.0006863 (PMC6199001; doi:10.1371/journal.pntd.0006863)
Supplement: S2 Fig — A. Protein description categories for transcripts significantly more abundant at peak parasitaemia than ascending parasitaemia. B. Protein description categories for transcripts with significantly reduced abundance at peak parasitaemia relative to ascending parasitaemia. (PDF) [file pntd.0006863.s008.pdf]

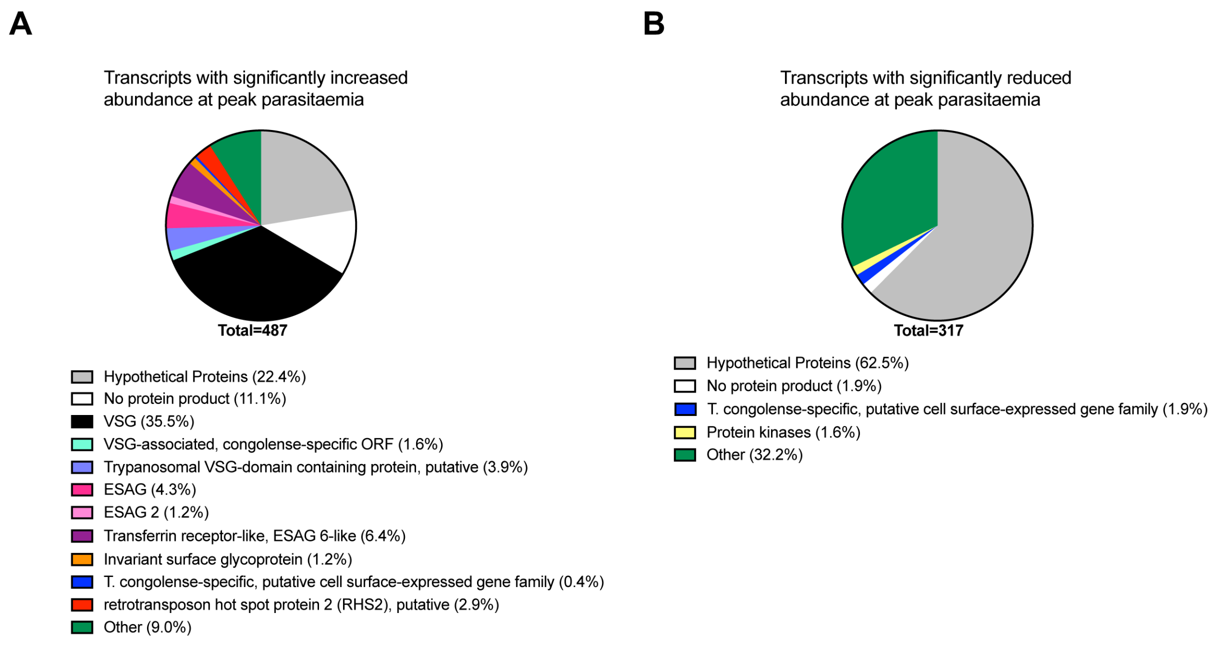
A
Transcripts with significantly increased abundance at peak parasitaemia
Total=487
Hypothetical Proteins (22.4%)
No protein product (11.1%)
VSG (35.5%)
VSG-associated, congolense-specific ORF (1.6%)
Trypanosomal VSG-domain containing protein, putative (3.9%)
ESAG (4.3%)
ESAG 2 (1.2%)
Transferrin receptor-like, ESAG 6-like (6.4%)
Invariant surface glycoprotein (1.2%)
T. congolense-specific, putative cell surface-expressed gene family (0.4%)
retrotransposon hot spot protein 2 (RHS2), putative (2.9%)
Other (9.0%)
B
Transcripts with significantly reduced abundance at peak parasitaemia
Total=317
Hypothetical Proteins (62.5%)
No protein product (1.9%)
T. congolense-specific, putative cell surface-expressed gene family (1.9%)
Protein kinases (1.6%)
Other (32.2%)
