# Supplementary figures and images for: A gene expression comparison of Trypanosoma brucei and Trypanosoma congolense in the bloodstream of the mammalian host reveals species-specific adaptations to density-dependent development
Source: PLoS Negl Trop Dis. 2018 Oct 11;12(10):e0006863. doi: 10.1371/journal.pntd.0006863 (PMC6199001; doi:10.1371/journal.pntd.0006863)

Surface phylome regulation (unique read mapping)

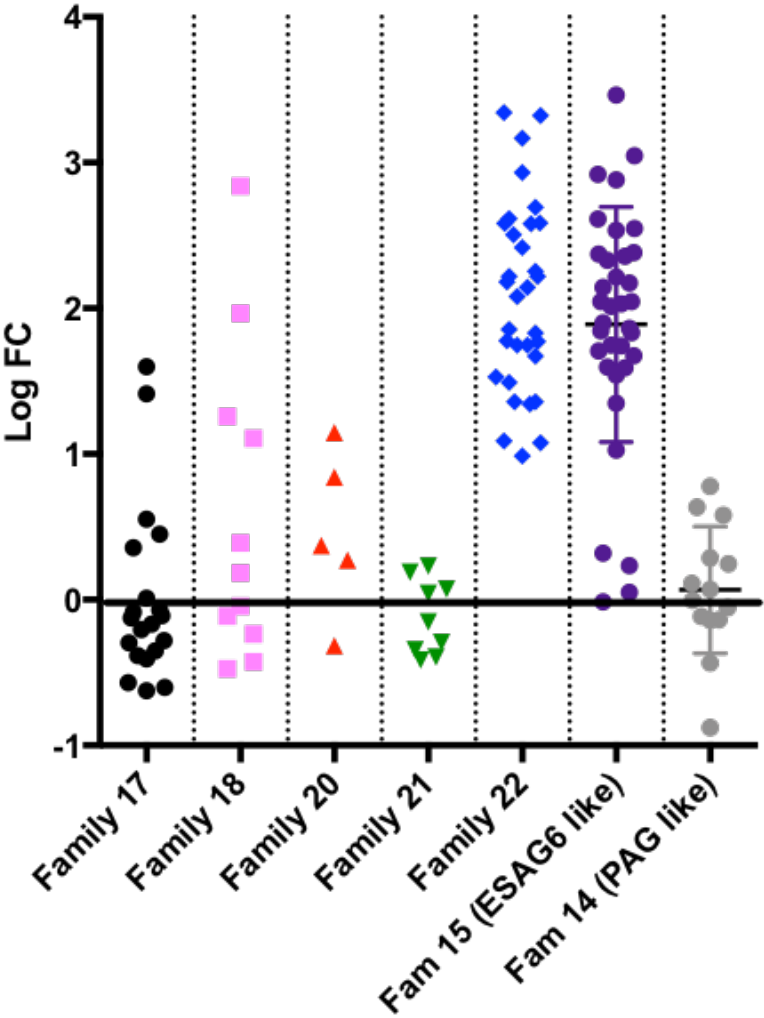

Supplement: S3 Fig — Analysis of the expression of surface phylome members that are unique to T. congolense. The graph shows assignment to different family members accounting only for unique reads specific to particular members of each family. (PDF) [file pntd.0006863.s009.pdf]

A

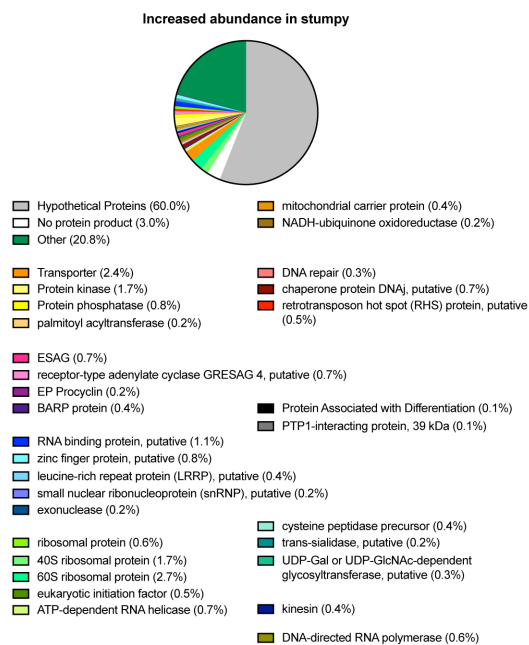

B

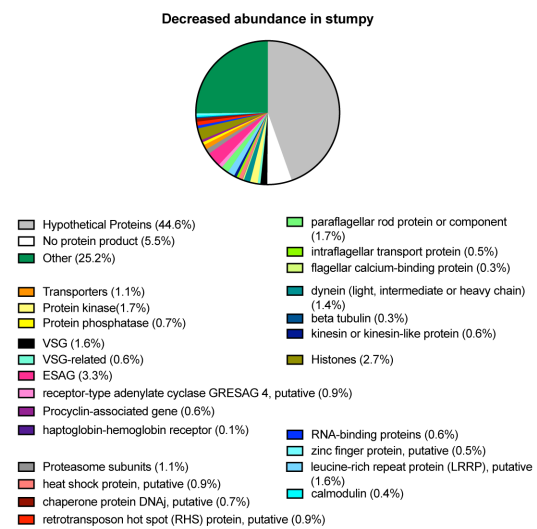

Supplement: S5 Fig — A. Transcripts with increased abundance in stumpy relative to slender parasites. B. Transcripts with decreased abundance in stumpy relative to slender parasites. (PDF) [file pntd.0006863.s011.pdf]
