# Supplementary material for: A gene expression comparison of Trypanosoma brucei and Trypanosoma congolense in the bloodstream of the mammalian host reveals species-specific adaptations to density-dependent development
Source: PLoS Negl Trop Dis. 2018 Oct 11;12(10):e0006863. doi: 10.1371/journal.pntd.0006863 (PMC6199001; doi:10.1371/journal.pntd.0006863)
Supplement: S4 Fig — The image shows the TritrypDB graphical representation of the BLAST hits returned using gene code TcIL3000_0_60190. The graphical representation was derived by running Wu Blast—BlastP against the NR database. (PDF) [file pntd.0006863.s010.pdf]

# TcIL3000_0_60190

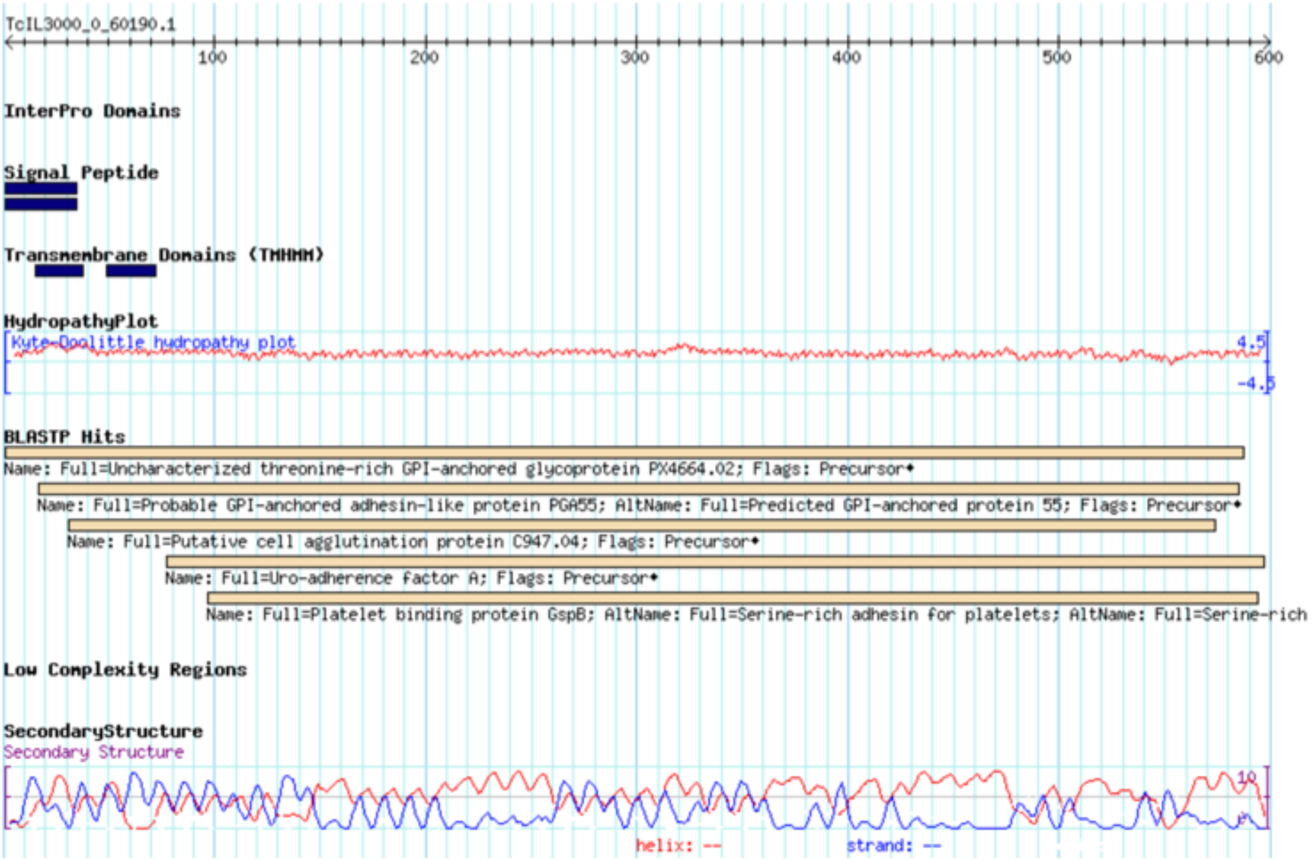


```
MTLSEVIPGFVKITLFEVISGFVIITLFEVLSGFVIITLFDVISGFVRITLFEVISGFVK
ITLFEVIPGFVKITLFEVISGFVKITLFEVISGFVKITLFEVISGFVKITLSEVISGYVK
ITLSEVISGFVKITLFEVISGYVKITLSEVISGFVKMTLSEVISGFVKITLSEVISGFVK
ITLSEVISGFVKITLSEVISGFVKITLSEVISGFVKMTLSEVISGFVEMTLFEVISGFVK
MTLFEVISGFVKMTLFEVISGFVKITLFEVISGFVKITLFEVISGFVKITLSEVISGFVK
ITLSEVISGFVKITLFEVILGFVGITLFEVISGFVKITLFEVISGFVKITLFEVISGFVK
ITLSEVISGFVRMTLSEIISGFVRITLSEVISGFVKITLSDVISGFVKMTLSEVISGFVK
ITLSEVISGFVKMTLFEVISGFVRMTLSEVISGFVKMTLSEVISGFVKMTLSAVLSGFVK
MTFSEVIPGFVRITLSEIISGCVRISLSEVILGFVKMTLSDVLSGFVKTTLSDVISGFVK
ITLSEAISGYVKITPSKVISGFVKMTLSAVISGYVKIALSEVISGFLKITLSEVISGFV
```
